# An artificial intelligence-assisted PARP-1-targeted PET imaging agent [^68^Ga]Ga-DOTA-FZPF: From bench to clinic

**DOI:** 10.1016/j.mtbio.2026.103728

**Published:** 2026-10-07

**Authors:** Mengjing Ji, Chunjuan Jiang, Yue Xi, Shuai Liu, Simin He, Jianping Zhang, Xiaoping Xu, Zhongyi Yang, Xiangwei Wang, Shaoli Song

**Affiliations:** a Department of Nuclear Medicine, Fudan University Shanghai Cancer Center, Shanghai, 200032, China; b Department of Oncology, Shanghai Medical College, Fudan University, Shanghai, 200032, China; c Center for Biomedical Imaging, Fudan University, Shanghai, 200032, China; d Shanghai Engineering Research Center of Molecular Imaging Probes, Shanghai, 200032, China

**Keywords:** Artificial intelligence, PARP, PET/CT imaging, Fuzuloparib, Radiotracer

## Abstract

Considering that clinical data for ^68^Ga-labeled poly(ADP-ribose) polymerase (PARP)-targeted probes have not yet been reported and integrated artificial intelligence (AI) workflows for radiotracer development are still lacking, to support radiotracer development and clinical translation, we constructed an AI-assisted multi-stage prioritization workflow and then synthesized two ^68^Ga-labeled PARP-1-targeted probes, validating their performance through *in vitro*, *in vivo*, and clinical evaluations. An AI-guided multi-objective workflow (encoder-decoder recurrent neural network (RNN) generation, convolutional neural network (CNN) docking scoring, Synthetic Complexity Learned from a Reaction Corpus (SCScore)-based tractability triage, and deep neural network (DNN)) was used to prioritize a derivatization-ready Fuzuloparib analog (NH_2_-FZPF) and to guide chelator installation, yielding DOTA-FZPF and NOTA-FZPF precursors for subsequent ^68^Ga radiolabeling. Both [^68^Ga]Ga-DOTA-FZPF and [^68^Ga]Ga-NOTA-FZPF exhibited high radiochemical purity, stability, and PARP-1-targeted specificity. In tumor models, [^68^Ga]Ga-DOTA-FZPF displayed favorable tumor uptake and rapid background clearance, supporting further evaluation. Exploratory positron emission tomography (PET) assessments of Fuzuloparib target engagement show the specificity and functionality of [^68^Ga]Ga-DOTA-FZPF, supporting *in vivo* evaluation of PARP inhibitor target engagement and pharmacodynamics. This study recruited 4 cancer patients and performed positron emission tomography/computed tomography (PET/CT) scans using [^68^Ga]Ga-DOTA-FZPF. Biopsy specimens showed that PET/CT-positive lesions corresponded to regions of high PARP-1 expression. This study demonstrates how an AI rational design workflow can support decision-making in radiotracer design while maintaining synthetic feasibility and translational potential. These preliminary findings support the clinical feasibility of [^68^Ga]Ga-DOTA-FZPF and suggest that it is promising for non-invasive assessment of PARP-1 expression and dynamic monitoring of *in vivo* target occupancy and pharmacodynamics.

## Highlights

• Established an end-to-end AI-aided platform for rational design of PARP-1 radiotracers.
• Designed the novel [^68^Ga]Ga-DOTA-FZPF PET tracer with favorable PARP-1 targeting capability and high tumor uptake.
• Preliminary clinical exploration of [^68^Ga]Ga-DOTA-FZPF was completed.
• This AI-assisted approach supports informed radiotracer design decisions and shows promising translational applications.

## Graphical abstract

**Figure:**
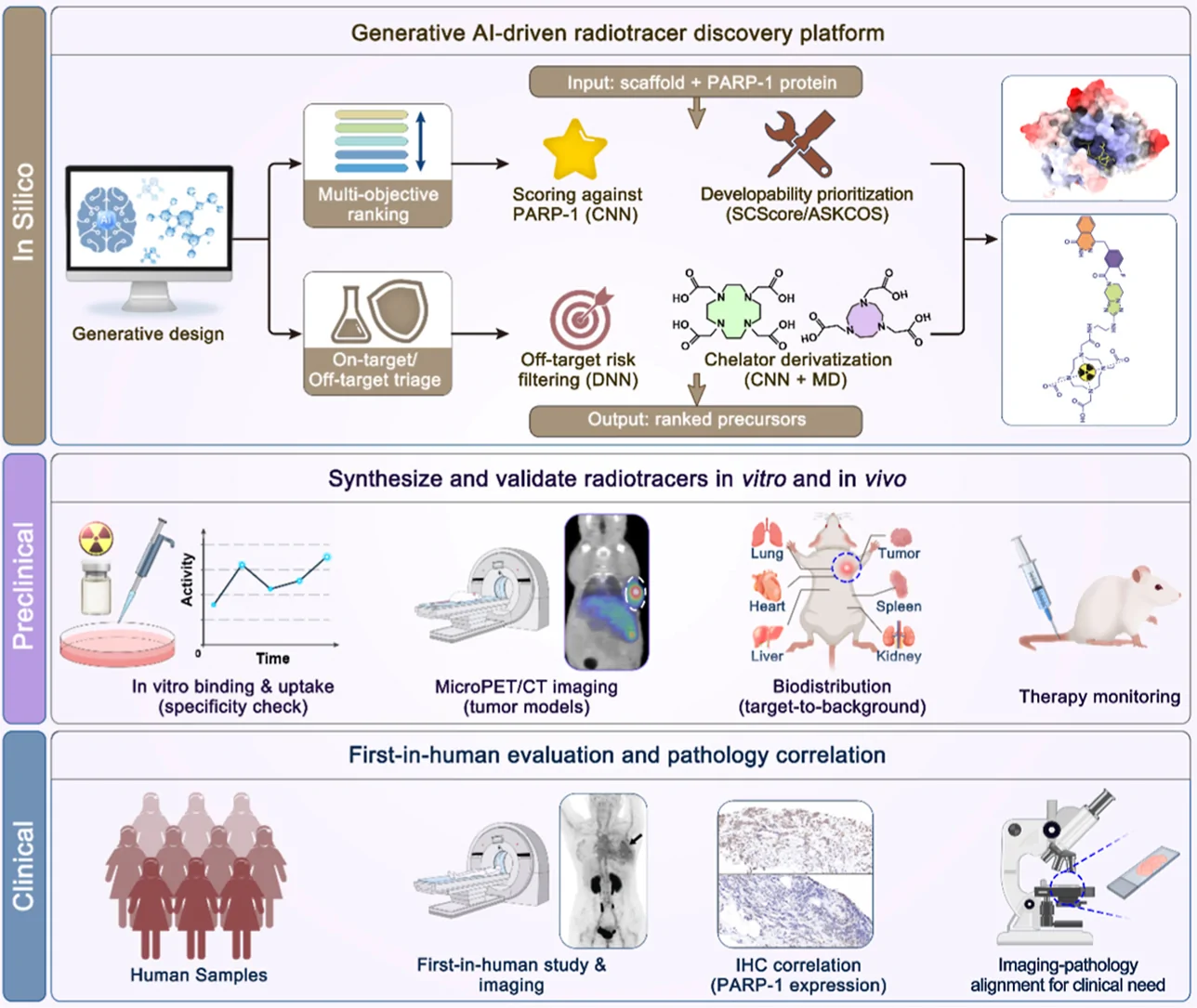

## 1 Introduction

Poly(ADP-ribose) polymerase (PARP) is a nucleoprotein involved in DNA damage detection and repair. Its inhibitors have demonstrated efficacy against multiple cancer types, including breast, ovarian, prostate, and small-cell lung cancers [[1], [2], [3], [4], [5]]. PARP comprises at least 17 distinct isoforms, with PARP-1 being the most abundantly expressed member of the family, accounting for over 90 % of DNA repair functions [6]. Due to the enhanced activity of PARP-1 resulting from dysregulated DNA damage repair pathways in various tumor cells, PARP-1 has emerged as a novel therapeutic and diagnostic target in cancer [7].

To date, various PARP inhibitors (PARPis), including Olaparib [7], Rucaparib [8], Niraparib [9], and Talazoparib [10], have received clinical approval from the U.S. Food and Drug Administration (FDA) and the European Medicines Agency (EMA) [11]. Given the prominent role of PARP inhibitors in cancer therapy, determining tumor PARP-1 expression levels may help predict sensitivity to PARP-1-targeted therapies. PARP imaging agents may be helpful in pre-treatment phases to guide response prediction and facilitate patient stratification. During the mid-treatment and post-treatment phases, they can quantify the tumor response to therapy.

Currently, pathological biopsy remains the gold standard for clinically assessing PARP protein expression levels. Due to tumor heterogeneity, biopsy results from a single lesion may not reflect the gene expression patterns in other metastatic sites. Furthermore, obtaining reliable, high-quality biopsy tissue from multiple disease sites (such as the lungs or brain) requires highly invasive and complex surgical procedures. These challenges have driven the development of noninvasive imaging protocols to assess overall disease severity and more effectively guide the selection of targeted therapies, including PARP inhibitors. Noninvasive molecular imaging represents a promising approach for visualizing lesions and quantitatively assessing PARP expression.

The need to characterize PARP-1 expression at the systemic level has driven the design and development of radiolabeled PARP inhibitor derivatives. This has enabled noninvasive assessment of PARP-1 expression using imaging modalities such as positron emission tomography (PET) and single-photon emission computed tomography (SPECT). Over the past decade, the synthesis of radiolabeled PARP-1-targeting imaging probes has accelerated. Moreover, reported compounds such as [^18^F]FTT and [^18^F]PARPi have undergone multiple clinical trials and demonstrated considerable clinical value [[12], [13], [14], [15], [16]].

As China's first PARP inhibitor with independent intellectual property rights, Fuzuloparib has expanded the number of PARP inhibitors available for cancer treatment [17]. Approved in China on December 11, 2020, Fuzuloparib demonstrates potent tumor cell killing activity (IC_50_ = 1.46 nM) [17]. This study structurally modified the clinically established PARP inhibitor Fuzuloparib to design and synthesize a novel gallium-68-labeled probe with high specificity for PARP.

The development of radiolabeled molecular imaging probes has long relied on experience-driven iterative optimization. In practice, the introduction of linkers/chelators and the regulation of *in vivo* pharmacokinetics often depend on trial-and-error adjustments, as minor structural alterations can significantly impact target binding, off-target profiles, plasma protein binding, and clearance pathways. Radiotracer development requires the joint consideration of target binding, synthetic feasibility, and *in vivo* behavior. Linker and chelator modifications introduce additional design choices that must be assessed through medicinal chemistry judgment and experimental evaluation.

To support these design decisions, we constructed and validated an AI-assisted multi-stage prioritization workflow tailored for radiotracer development. This workflow integrates generative molecular design with structure-aware multi-objective screening strategies to support the design and prioritization of PARP-1-targeting probes. Using Fuzuloparib as the lead scaffold, we applied a SMILES-based conditional chemical language model (recurrent neural network, RNN) to generate candidate structures [18]. Subsequently, a convolutional neural network (CNN)-guided conformation assessment predicted their binding affinity to PARP-1 [19]. Concurrently, a synthesis complexity model (SCScore) trained on reaction data stratified candidates by synthesizability, enabling joint “affinity-synthesizability” decision-making [20]. The core inhibitor (NH_2_-FZPF) was further converted into a DOTA/NOTA-conjugated radiotracer precursor. CNN scoring suggested binding poses and protein–ligand interactions, followed by explicit-solvent molecular dynamics simulations to assess complex stability. Simultaneously, a deep learning reverse-screening module (DNN target prediction) was introduced to characterize potential multi-target effects and to proactively mitigate off-target risks systematically [21]. Overall, this workflow from AI-generated structures to experimentally verifiable radiotracers addresses a critical gap in the field.

Based on this workflow, we developed the PARP-1-targeting radiotracer [^68^Ga]Ga-DOTA-FZPF. This study advanced it from preclinical proof of concept to an exploratory first-in-human PET/CT evaluation, while providing previously unavailable clinical observations for a^68^Ga-labeled PARP-1-targeted probe. We evaluated its PARP-1-associated uptake, preclinical imaging characteristics, capacity to report inhibitor target engagement, and preliminary human tolerability and imaging feasibility.

## 2 Results

### 2.1 Scaffold-constrained Lib-INVENT decoration generated a focused fuzuloparib analog set

To explore derivatization-compatible substitutions while retaining the pharmacophore-containing core of Fuzuloparib, two scaffold-constrained decoration runs were performed using the pretrained Lib-INVENT reaction-based decorator model. In the first run, the variable substituent was attached directly to the triazole-containing ring system, yielding compounds 004–007. In the second run, a methylene-extended attachment-point definition was used, yielding compounds 001–003. Together, the two runs generated a compact set of seven analogs with methyl, ethyl, propyl, amino, aminomethyl, trifluoromethyl, or phenyl substituents (Fig. S1).

The SCScore values ranged from 4.6 to 4.8, with compound 005 (NH_2_-FZPF) receiving the lowest score (4.6), corresponding to the lowest model-predicted synthetic complexity within this set. Moreover, compound 005 was advanced primarily also because of its derivatization-ready amine and the commercial availability of a key amine-containing building block. Its docking results did not indicate an obviously incompatible binding orientation within the PARP-1 pocket (Table S1).

### 2.2 CNN-guided docking of the ^nat^Ga-complexed probes with PARP-1

Docking of [^nat^Ga]Ga-NOTA-FZPF and [^nat^Ga]Ga-DOTA-FZPF with PARP-1 (PDB ID: 5DS3) showed that Fuzuloparib-derived cores of both gallium-containing ligands occupied the same general binding pocket as the parent compound Fuzuloparib, while the linker and chelator-containing regions adopted distinct orientations (Fig. 1A–C). The three molecules shared interactions with key residues, including S904 and G863, while additional local residue environments varied among the complexes, indicating ligand-dependent binding configurations within the modeled complexes (Fig. 1D). Moreover, the corresponding docking metrics are summarized in Table S1.

**Fig. 1:**
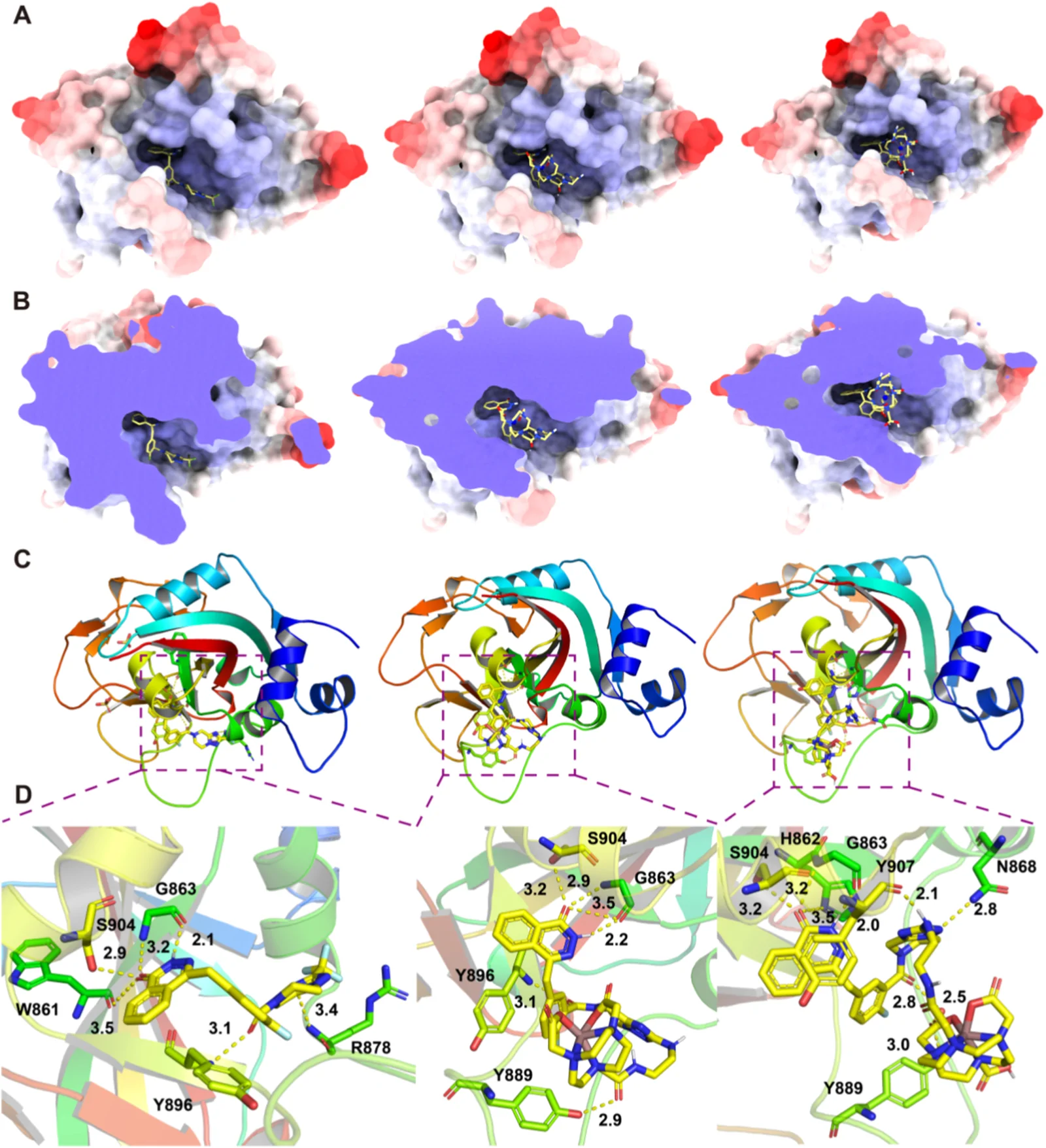
Binding modes of the compounds Fuzuloparib, [^nat^Ga]Ga-NOTA-FZPF, and [^nat^Ga]Ga-DOTA-FZPF with PARP-1 (PDB ID: 5DS3). (A) Predicted docked binding poses of Fuzuloparib (Left), [^nat^Ga]Ga-NOTA-FZPF (Center), and [^nat^Ga]Ga-DOTA-FZPF (Right) bound to the domain of PARP-1. (B) Cross-section structures of Fuzuloparib (Left), [^nat^Ga]Ga-NOTA-FZPF (Center), and [^nat^Ga]Ga-DOTA-FZPF (Right) bound to the domain of PARP-1. (C) Binding modes of Fuzuloparib (Left), [^nat^Ga]Ga-NOTA-FZPF (Center), and [^nat^Ga]Ga-DOTA-FZPF (Right) bound to the domain of PARP-1 (Left). (D) H-bond interactions between Fuzuloparib (Left), [^nat^Ga]Ga-NOTA-FZPF (Center), and [^nat^Ga]Ga-DOTA-FZPF (Right) bound to the domain of PARP-1.

### *2.3* Deep neural network (*DNN) target prediction*

To evaluate whether functionalization and the introduction of chelating groups maintain preferential targeting toward the target protein, we employed a DNN based on the molecular fingerprint ECFP4+MHFP6 for AI-based target prediction. Results indicate that Fuzuloparib, NH_2_-FZPF, NOTA-FZPF, and DOTA-FZPF all rank PARP-1 as their top predicted target (rank = 1) with high confidence (0.98 - 1.00), annotated as an enzyme/transferase belonging to a single human protein (Table S2).

### 2.4 Molecular dynamics

In both systems, the protein backbone RMSD converged, and the radius of gyration remained stable throughout the 100-ns simulations. Compared with Fuzuloparib, DOTA-FZPF exhibited greater ligand RMSD fluctuations, whereas the overall complex RMSD remained relatively stable (Fig. 2A and B).

**Fig. 2:**
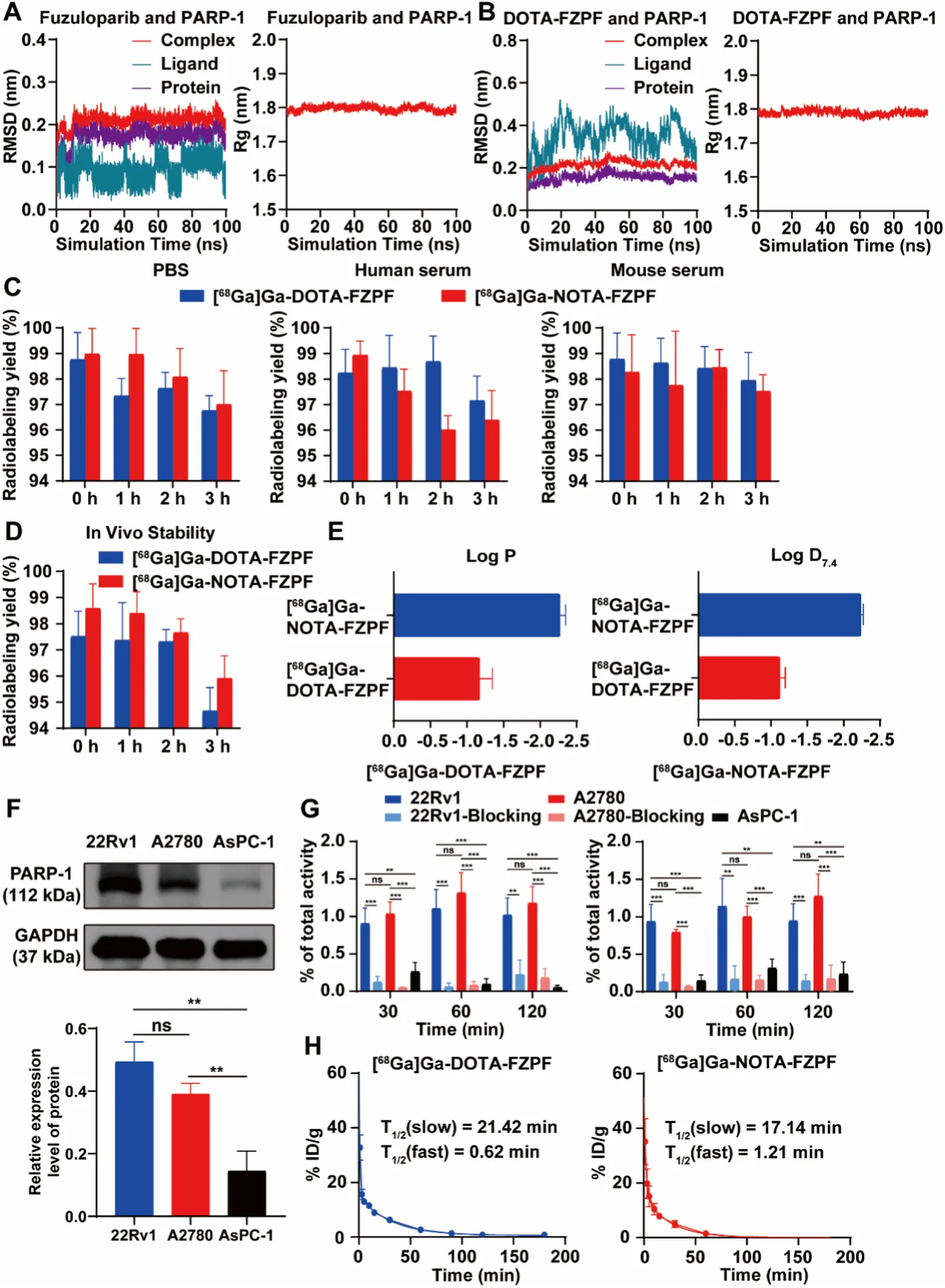
Comprehensive characterization of PARP-1-targeted radiotracers: molecular dynamics, stability, solubility, cellular uptake and pharmacokinetics. (A) RMSD (Left) and distance (Right) analyses of the Fuzuloparib-PARP-1 complex over a 100 ns simulation using GROMACS; (B) RMSD (Left) and distance (Right) analyses of the DOTA-FZPF-PARP-1 complex over the same simulation period. (C) Stability of the radiotracers *in vitro*. (saline, human serum, and mouse serum) (1, 2, and 3 h). (D) Stability of the radiotracers *in vivo*. (1, 2, and 3 h). (E) Solubility for two radiotracers. (F) PARP-1 expression in AsPC-1, A2780, and 22Rv1 cell lines. (G) Cell uptake of [^68^Ga]Ga-DOTA-FZPF (Left) and [^68^Ga]Ga-NOTA-FZPF (Right). (H) Blood clearance curves of [^68^Ga]Ga-DOTA-FZPF (Left) and [^68^Ga]Ga-NOTA-FZPF (Right).

### 2.5 Radiochemistry

Gallium-68-labeled radiopharmaceuticals have demonstrated promising applications in PET imaging [22]. High-quality images can be obtained within approximately 1 h after intravenous injection.

The two precursors were structurally confirmed by ^1^H NMR and mass spectrometry (Figs. S6–S9) in the Supplementary Material. The chemical structures of the two gallium-68-labeled radiotracers are shown in Fig. S2A. The radiochemical purities (RCPs) of [^68^Ga]Ga-DOTA-FZPF and [^68^Ga]Ga-NOTA-FZPF were confirmed by radio-HPLC as 99.35 ± 0.56 % and 98.48 ± 1.22 %, respectively (Fig. S2B). The retention times of both radiotracers on radio-HPLC were determined (radioactive peaks: [^68^Ga]Ga-DOTA-FZPF, 7.70 min; [^68^Ga]Ga-NOTA-FZPF, 7.71 min) (Fig. S2D). For the precursors, UV peaks were observed at 7.52 min for DOTA-FZPF and 8.08 min for NOTA-FZPF (Fig. S2C). These similar retention times indicate successful radiolabeling of the precursors with gallium-68. The molar activities of [^68^Ga]Ga-DOTA-FZPF and [^68^Ga]Ga-NOTA-FZPF were 7.89–10.69 and 7.10–10.09 GBq/μmol, respectively.

### 2.6 Distribution coefficients

This study evaluates hydrophilicity using the distribution of gallium-68-labeled radiotracers between the aqueous phase (PBS or ultrapure water) and the organic phase (1-octanol). As shown in Fig. 2E, the Log P and Log D_7.4_ values for [^68^Ga]Ga-DOTA-FZPF were determined to be −1.11 ± 0.23 and −1.15 ± 0.05, respectively, while those for [^68^Ga]Ga-NOTA-FZPF were −2.28 ± 0.11 and −2.23 ± 0.04. These results indicate that both radiopharmaceuticals are highly hydrophilic, with [^68^Ga]Ga-NOTA-FZPF exhibiting the greatest hydrophilicity.

### 2.7 Stability of the radiotracers

After incubation for 180 min in physiological saline, mouse serum, and human serum, [^68^Ga]Ga-DOTA-FZPF and [^68^Ga]Ga-NOTA-FZPF retained over 95 % (Fig. 2C). Urine samples were collected from mice at 3 h post-injection for radio-HPLC analysis. Fig. 2D shows that the radiotracers maintained high stability (>95 %) after 3 h of *in vivo* metabolism.

### 2.8 Cellular uptake

To validate the targeting capability of the radiotracers toward PARP-1, we conducted cellular experiments using 22Rv1 and A2780 (high PARP-1 expression) and AsPC-1 (low PARP-1 expression) cell lines (Fig. 2G). Western blot analysis revealed significantly higher PARP-1 expression levels in 22Rv1 and A2780 cell lines than in the AsPC-1 cell line (22Rv1 vs. AsPC-1: *P* = 0.003; A2780 vs. AsPC-1: *P* = 0.004) (Fig. 2F). To assess the specificity of [^68^Ga]Ga-DOTA-FZPF and [^68^Ga]Ga-NOTA-FZPF, we measured their uptake in these two cell lines. At 30, 60, and 120 min of incubation, 22Rv1 and A2780 cell lines had significantly higher uptake of [^68^Ga]Ga-DOTA-FZPF or [^68^Ga]Ga-NOTA-FZPF than the AsPC-1 cell line, consistent with the Western blot results (The *P*-values were all less than 0.05) (Fig. 2G). Blocking experiments showed that co-incubation with an excess (500-fold) of the lead compound significantly reduced uptake in both 22Rv1 and A2780 cell lines (All *P* < 0.05) (Fig. 2G).

### 2.9 Pharmacokinetics

We determined the pharmacokinetic parameters of the radiotracers in normal mice to assess drug metabolism *in vivo*. As shown in Fig. 2H, the distribution half-life (t_1/2_(fast)) for both gallium-68-labeled radiotracers was less than 2 min ([^68^Ga]Ga-DOTA-FZPF: 0.62 min; [^68^Ga]Ga-NOTA-FZPF: 1.21 min). The elimination half-lives (t_1/2_(slow)) were 21.42 and 17.14 min, respectively.

### 2.10 Acute tolerability at tracer dose

To obtain a preliminary assessment of the short-term tolerability of the radiotracers at the administered tracer dose, normal mice were monitored for 7 days after intravenous injection of [^68^Ga]Ga-DOTA-FZPF or [^68^Ga]Ga-NOTA-FZPF. No hematological parameters or serum biochemical markers of hepatic and renal function were observed compared with the saline control group (Fig. S3A). H&E staining of the heart, liver, spleen, lung, and kidney revealed no apparent histopathological abnormalities at 7 days post-injection (Fig. S3B). These findings support the preliminary acute tolerability of the two radiotracers under the tested tracer-dose conditions.

### 2.11 Micro-PET/CT imaging and ex vivo biodistribution evaluation of [^68^Ga]Ga-DOTA-FZPF and [^68^Ga]Ga-NOTA-FZPF in 22Rv1, A2780, and AsPC-1 tumor models

The *in vivo* tumor-targeting capability of gallium-68-labeled radiotracers was investigated in tumor-bearing mice using micro-PET/CT (Fig. 3, Fig. 5 and S4). Mice bearing 22Rv1, A2780, or AsPC-1 tumors received 5.55 - 7.40 MBq of [^68^Ga]Ga-DOTA-FZPF and [^68^Ga]Ga-NOTA-FZPF via tail vein injection, followed by quantitative micro-PET/CT imaging at 30, 60, and 120 min post-injection. For [^68^Ga]Ga-DOTA-FZPF, significant radioactive concentrations were detected in tumors at 30, 60, and 120 min in PARP-1 high-expressing 22Rv1-and A2780-tumor-bearing mice (Fig. 3A, B, 3D, and 3E). Over time, [^68^Ga]Ga-DOTA-FZPF maintained high accumulation in tumor regions while gradually decreasing uptake in non-tumor areas such as the heart, liver, and intestine. In AsPC-1 tumor models with lower PARP-1 expression, [^68^Ga]Ga-DOTA-FZPF showed reduced uptake and shorter retention time (Fig. 3C and F). The accumulation of radioactivity in tumors after 60 min of [^68^Ga]Ga-DOTA-FZPF administration was most pronounced in 22Rv1 and A2780 tumor models, reaching 5.03 ± 0.77 %ID/g and 5.27 ± 0.40 %ID/g, respectively, significantly higher than the control AsPC-1 uptake of 1.90 ± 0.10 %ID/g (22Rv1 vs. AsPC-1, *P* = 0.002; A2780 vs. AsPC-1, *P* = 0.0002) (Fig. 3G). The results are consistent with the findings of PARP-1 immunohistochemistry staining (Fig. 3K). The tumor-to-muscle ratios (TMR) for [^68^Ga]Ga-DOTA-FZPF at 60 min in the 22Rv1, A2780, and AsPC-1 tumor-bearing mice were 14.80 ± 8.80, 6.79 ± 1.39, and 1.68 ± 0.16, respectively (Fig. 3H–J). Blockade experiments were performed in 22Rv1 and A2780 tumor-bearing mice expressing high levels of PARP-1. Tumor uptake in the blockade group was significantly lower than in the experimental group (At 60 min, 22Rv1: 5.03 ± 0.77 %ID/g vs. 1.53 ± 0.15 %ID/g, *P* = 0.003; A2780: 5.27 ± 0.40 %ID/g vs. 1.83 ± 0.31 %ID/g, *P* = 0.0003) (Fig. 3D and E). In Fig. 5 and S4, for [^68^Ga]Ga-NOTA-FZPF, significant radioactive concentrations were detected in tumors at 60 min in both 22Rv1 and A2780 tumor-bearing mice (2.77 ± 0.51 %ID/g and 3.40 ± 0.66 %ID/g). In AsPC-1 tumor-bearing mice with lower PARP-1 expression, [^68^Ga]Ga-NOTA-FZPF uptake was low (60 min, 1.60 ± 0.53 %ID/g), and retention time was shorter. The 22Rv1 model with high PARP-1 expression was selected to perform comparative imaging analysis (Fig. 5D–F) and biodistribution (Fig. 5G–H). Compared with [^68^Ga]Ga-NOTA-FZPF, [^68^Ga]Ga-DOTA-FZPF exhibited higher tumor accumulation and lower non-target organ uptake (60min: [^68^Ga]Ga-DOTA-FZPF vs. [^68^Ga]Ga-NOTA-FZPF, tumor, 5.03 ± 0.77 %ID/g vs. 2.77 ± 0.51 %ID/g, *P* = 0.014, Fig. 5E; tumor, 3.77 ± 0.56 %ID/g vs. 2.40 ± 0.31 %ID/g, *P* = 0.044, Fig. 5G), along with an improved TMR and T/NT (Fig. 5F and H). Notably, both micro-PET/CT imaging and ex vivo biodistribution results demonstrated that [^68^Ga]Ga-NOTA-FZPF exhibited high uptake in major normal organs, particularly the liver (60min: [^68^Ga]Ga-DOTA-FZPF vs. [^68^Ga]Ga-NOTA-FZPF, 2.43 ± 0.47 %ID/g vs. 7.27 ± 2.00 %ID/g, Fig. 5E; 2.84 ± 0.87 %ID/g vs. 12.39 ± 2.68 %ID/g, Fig. 5G), spleen(60min, [^68^Ga]Ga-DOTA-FZPF vs. [^68^Ga]Ga-NOTA-FZPF, 0.57 ± 0.18 %ID/g vs. 2.64 ± 1.21 %ID/g, Fig. 5G), and kidney (60min: [^68^Ga]Ga-DOTA-FZPF vs. [^68^Ga]Ga-NOTA-FZPF, 3.37 ± 0.42 %ID/g vs. 6.90 ± 3.61 %ID/g, Fig. 5E; 1.79 ± 0.34 %ID/g vs. 9.83 ± 1.26 %ID/g, Fig. 5G).

**Fig. 3:**
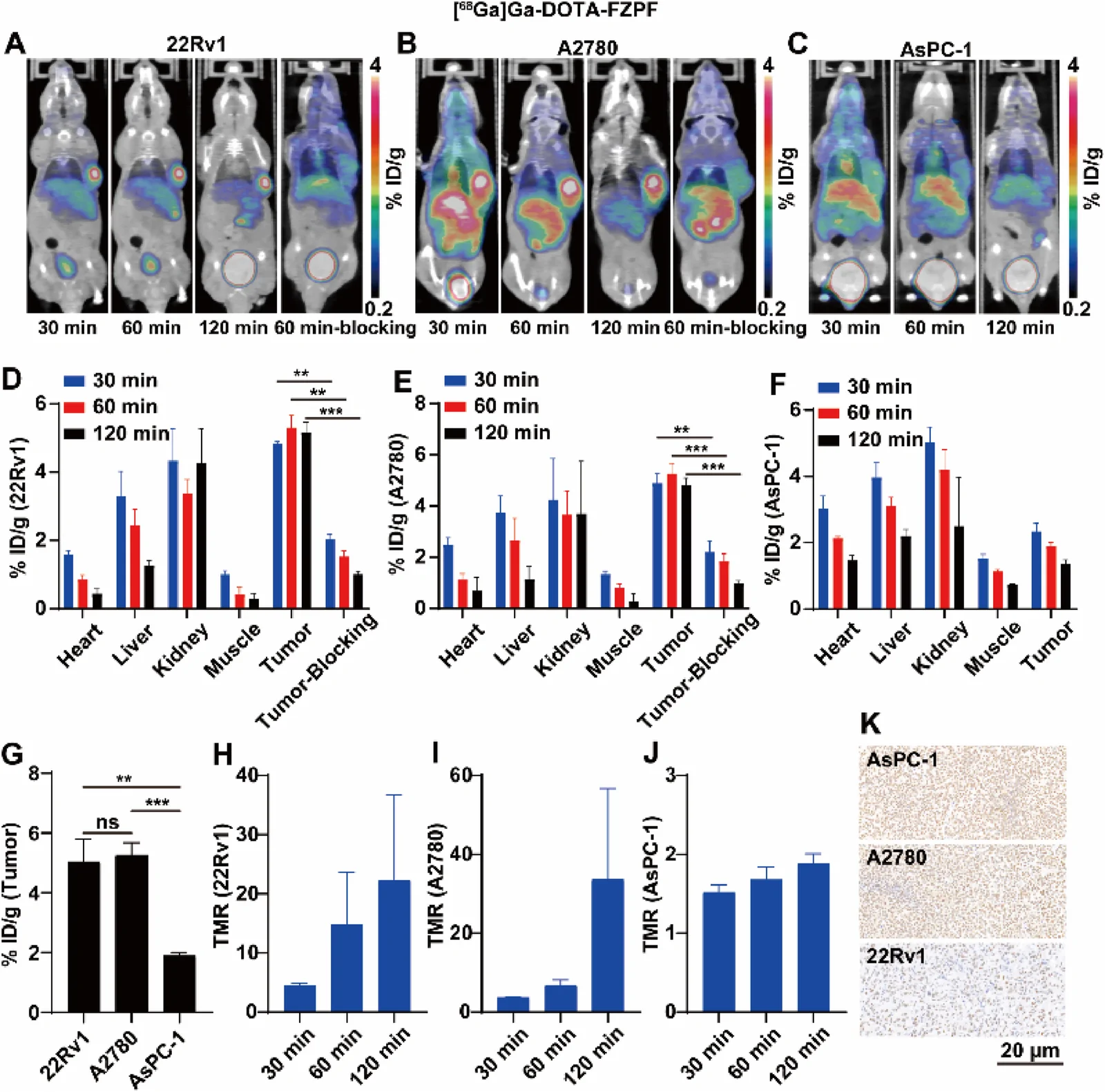
Preclinical evaluation of [^68^Ga]Ga-DOTA-FZPF radiotracer by micro-PET/CT imaging of AsPC-1, A2780, and 22Rv1 tumor models and immunohistochemistry staining of tumor slices. (n = 3) (A-C) Micro-PET/CT imaging of [^68^Ga]Ga-DOTA-FZPF in AsPC-1, A2780, and 22Rv1 tumor models. (D-F) Quantification of the regions of interest (ROIs) in the AsPC-1, A2780, and 22Rv1 tumor models expressed as %ID/g. (G) The Comparison of tumor uptake values in AsPC-1, A2780, and 22Rv1 tumor models 60 min after [^68^Ga]Ga-DOTA-FZPF injection. (H-J) The tumor-to-muscle ratios in the AsPC-1, A2780, and 22Rv1 model. (K) Immunohistochemistry staining of tumor slices of AsPC-1, A2780, and 22Rv1 tumor models.

The biodistribution of [^68^Ga]Ga-DOTA-FZPF in tumors and various organs across groups is shown in Fig. 4. The 22Rv1 and A2780 tumors exhibited significantly higher [^68^Ga]Ga-DOTA-FZPF accumulation (60 min, 3.77 ± 0.56 %ID/g and 6.51 ± 1.29 %ID/g) compared to the AsPC-1 group (60 min, 1.29 ± 0.33 %ID/g) (22Rv1 vs. AsPC-1, *P* = 0.003; A2780 vs. AsPC-1, *P* = 0.002). Notably, 60 min after administration, the TMR, tumor-to-blood ratio (TBR), and tumor-to-liver ratios (TLR) values for 22Rv1 and A2780 tumors were 15.36 ± 8.28 and 18.92 ± 5.31, 4.86 ± 0.35 and 3.64 ± 1.37, and 1.38 ± 0.28 and 2.14 ± 0.28, respectively.

**Fig. 4:**
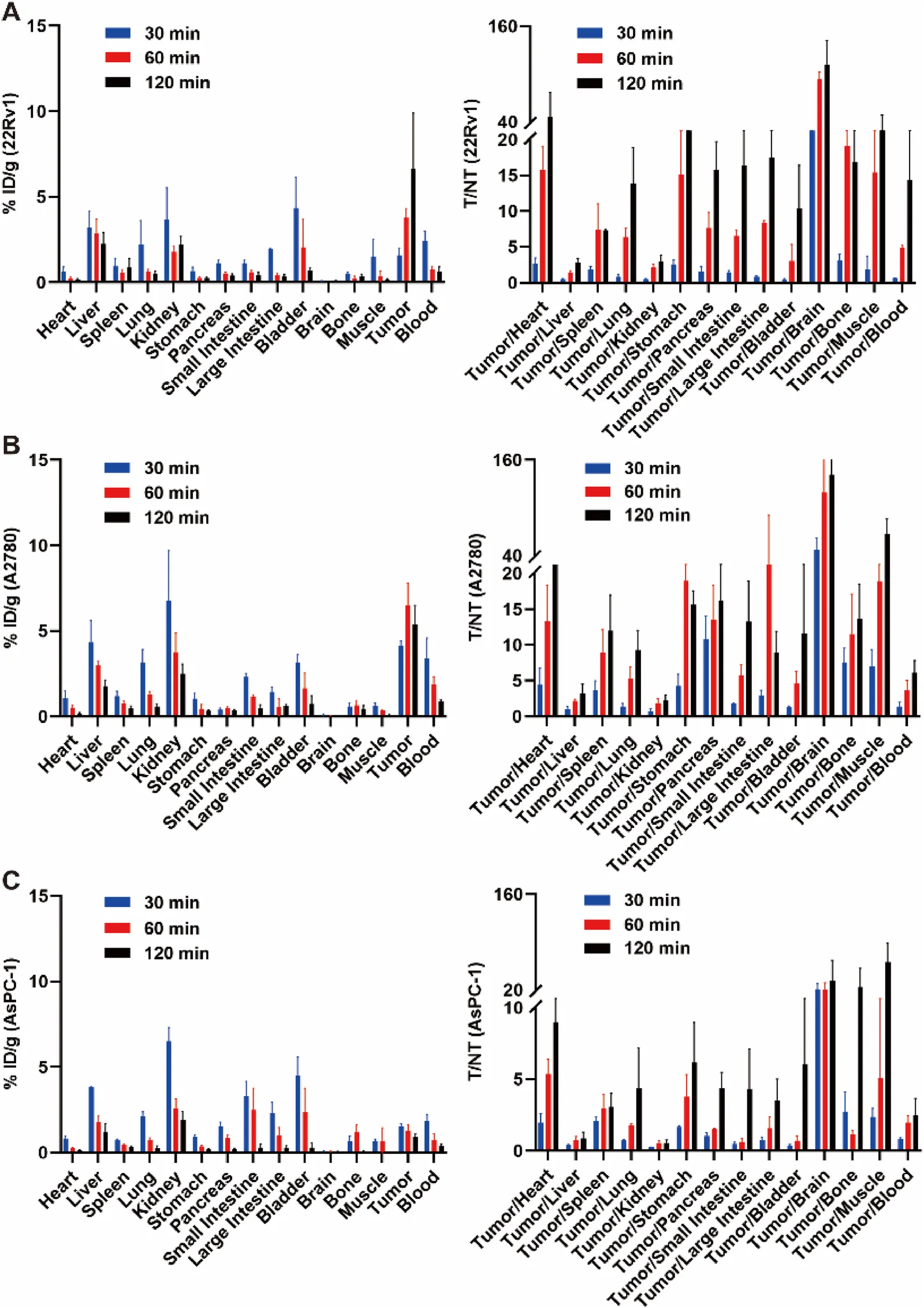
Biodistribution of [^68^Ga]Ga-DOTA-FZPF in tumor-bearing mice. (n = 3) (A) Biodistribution and the ratios of tumor-to-nontarget tissue in 22Rv1 tumor models. (B) Biodistribution and the ratios of tumor-to-nontarget tissue in A2780 tumor models. (C) Biodistribution and the ratios of tumor-to-nontarget tissue in AsPC-1 tumor models.

**Fig. 5:**
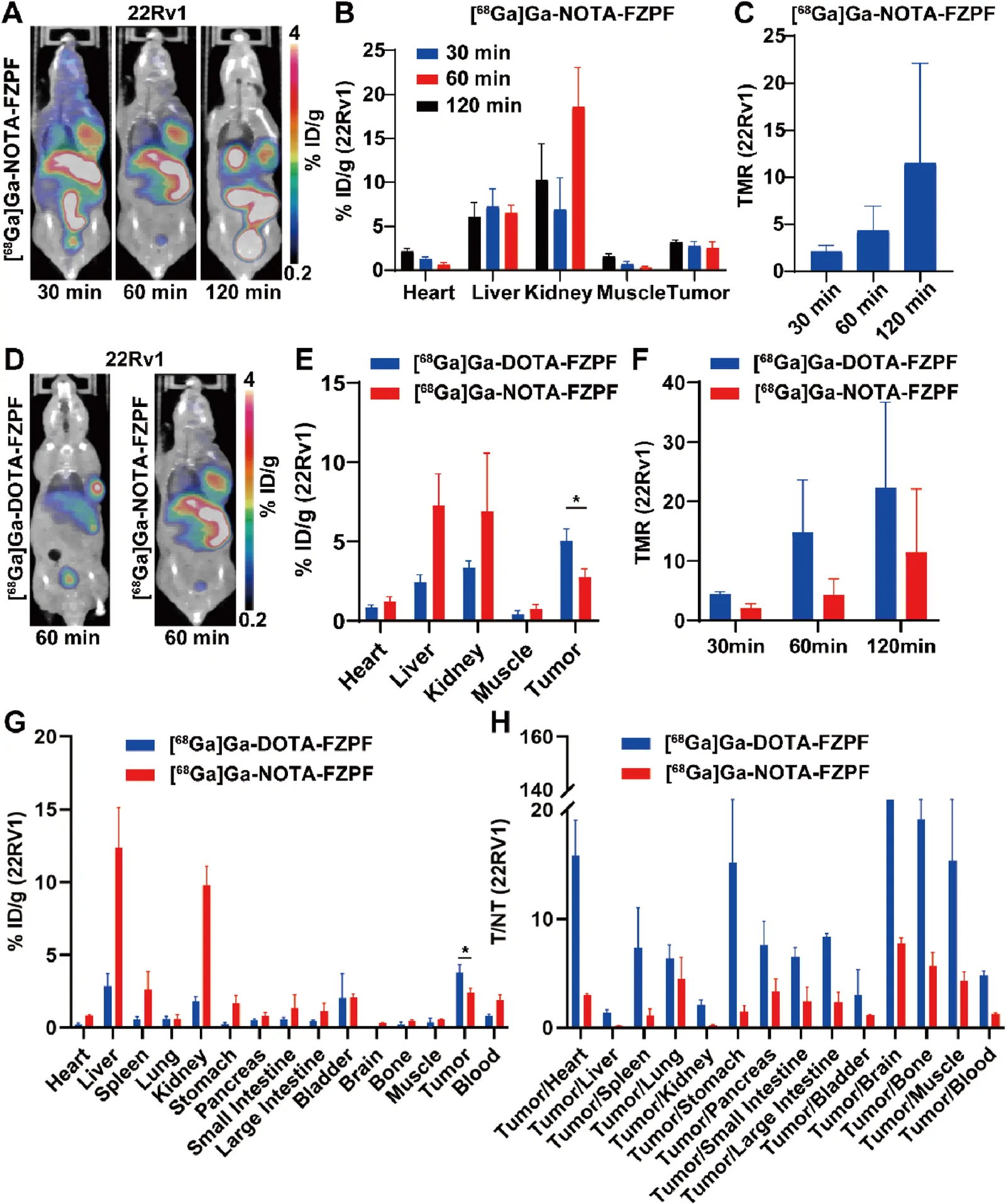
Diagnostic evaluation of [^68^Ga]Ga-NOTA-FZPF and comparative assessment of [^68^Ga]Ga-DOTA-FZPF and [^68^Ga]Ga-NOTA-FZPF in 22Rv1 model. MicroPET/CT imaging (A), quantification of regions of interest (B), and the tumor-to-muscle ratio (C) of [^68^Ga]Ga-NOTA-FZPF in 22Rv1 models. MicroPET/CT images (D) and quantification of regions of interest (E) of [^68^Ga]Ga-DOTA-FZPF and [^68^Ga]Ga-NOTA-FZPF at 60 min post-injection, and tumor-to-muscle ratios (F) at 30, 60, and 120 min post-injection, in 22Rv1 models. Biodistribution of major organs and tumor uptake (G) and Tumor-to-nontarget tissue ratios (H) at 60 min post-injection in 22Rv1 models.

Collectively, the higher uptake in PARP-1-high models and the reduction observed in blocking experiments were consistent with PARP-1-associated tumor uptake. Therefore, it was selected for subsequent clinical studies.

### 2.12 PET assessment of fuzuloparib target engagement

Tumor volume and body weight showed only modest differences between the Fuzuloparib-treated and control groups. PARP-1 IHC staining intensity was similar between groups at day 21, whereas [^68^Ga]Ga-DOTA-FZPF tumor uptake decreased during Fuzuloparib administration (Fig. 6B–F).

**Fig. 6:**
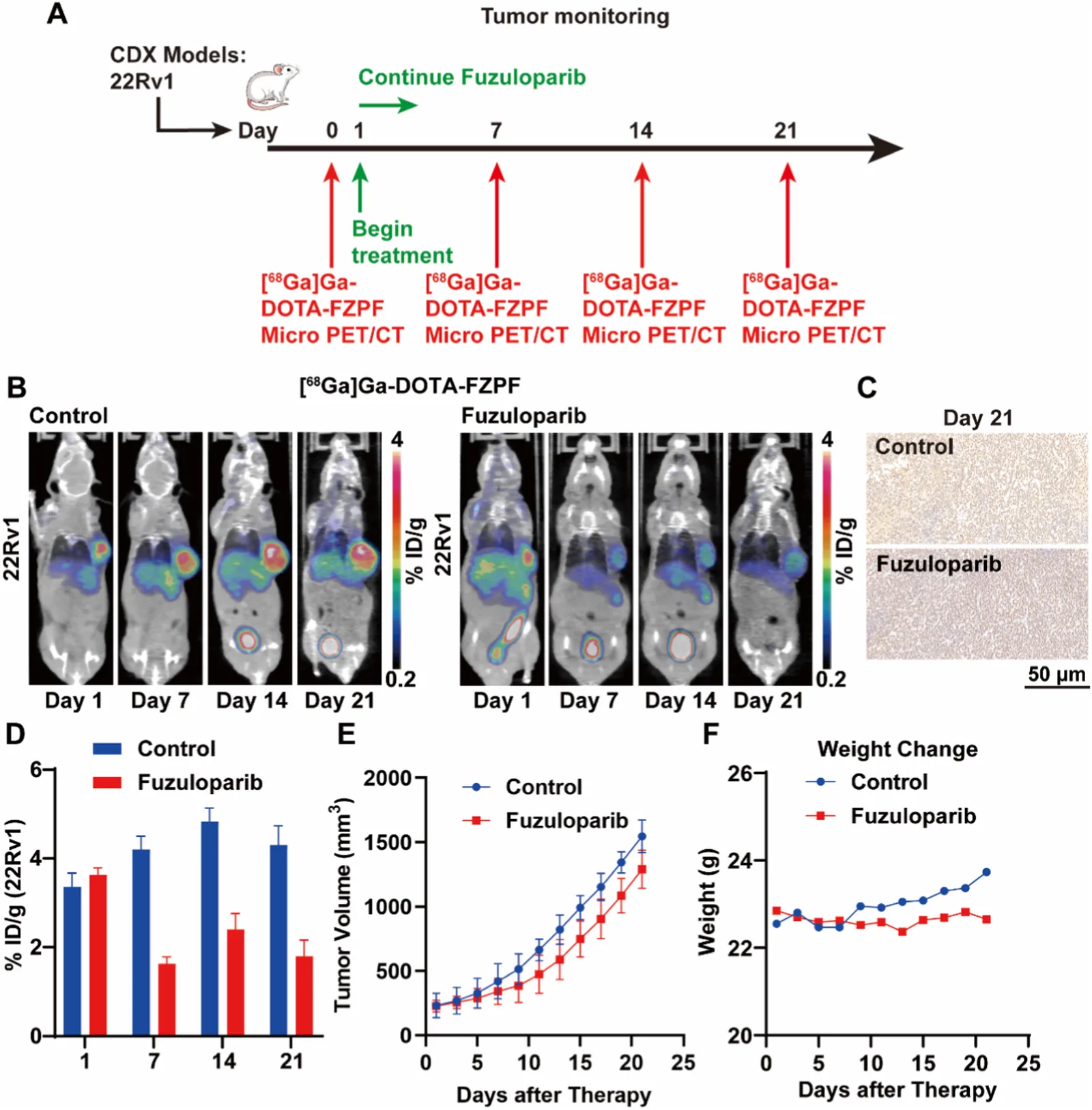
Serial PET assessment of Fuzuloparib-mediated PARP target engagement in CDX 22Rv1 tumor models. (n = 6) (A) Schematic of serial [^68^Ga]Ga-DOTA-FZPF micro-PET/CT imaging during repeated Fuzuloparib administration in 22Rv1 tumor-bearing mice. PET/CT imaging was performed at baseline and on days 1, 7, 14, and 21. (B) Representative [^68^Ga]Ga-DOTA-FZPF-PET/CT imaging pre- (control) and post-treatment (Fuzuloparib) (left and right, respectively). (C) Immunohistochemistry staining of the tumor slices pre- and post-treatment (top and bottom, respectively). (D) Tumor uptake values pre- and post-treatment. (E) Tumor volume data over time. (F) Weight Change over time.

### 2.13 First-in-human PET/CT imaging

Based on the favorable research results from the aforementioned experiments, we initiated a preliminary clinical study of [^68^Ga]Ga-DOTA-FZPF in cancer patients (approval number: 2503316-22). To date, the study has enrolled 3 breast cancer patients and 1 ovarian cancer patient. Because this was a small, exploratory first-in-human cohort, the clinical objective was to evaluate initial tolerability, imaging feasibility, and descriptive concordance between imaging and pathology.

Patient 1 was diagnosed with left breast cancer. [^68^Ga]Ga-DOTA-FZPF PET/CT imaging revealed increased uptake in the affected breast compared to the contralateral normal breast (SUVmax = 3.7 (left)/1.8 (right), Fig. 7A). Patient 2 presented with liver recurrence following a radical mastectomy for breast cancer. Clinical findings suggested multiple hepatic metastases, leading to a liver biopsy. [^68^Ga]Ga-DOTA-FZPF PET/CT imaging revealed no significant abnormal uptake throughout the body. Among these, the radioactive uptake in hepatic metastases was close to that of normal liver tissue (SUVmax = 1.3 (hepatic metastasis)/1.1 (normal liver), Fig. 7B). Immunohistochemistry results are shown in Fig. 7. Patient 1 exhibited high PARP-1 expression levels in the lesion. Patient 2 showed low PARP-1 expression levels in the lesion. Detailed [^68^Ga]Ga-DOTA-FZPF PET/CT uptake data for the four patients are provided in Fig. S5 and Tables S3–4.

**Fig. 7:**
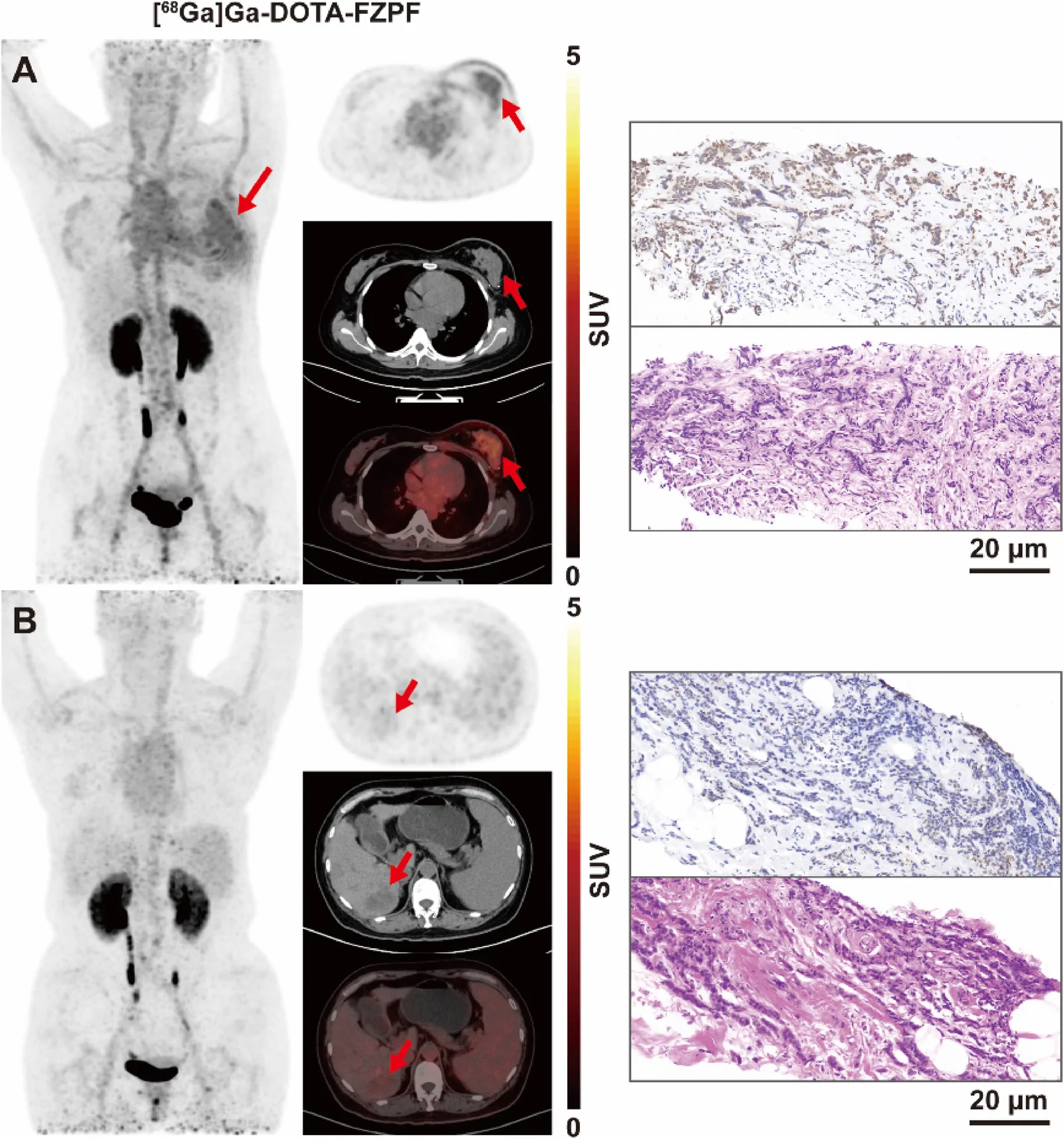
PET/CT images, PARP-1 IHC staining, and HE staining in representative breast cancer patients. A. [^68^Ga]Ga-DOTA-FZPF PET/CT shows high uptake in the left breast of Patient 1 (SUVmax = 3.7) (arrow). Pathology confirmed Patient 1 with left breast cancer. PARP-1 IHC staining of the breast lesion demonstrated high expression of PARP-1 protein in tumor cells. B. Patient 2 developed hepatic metastases following a radical mastectomy for breast cancer. [^68^Ga]Ga-DOTA-FZPF PET/CT revealed radioactive uptake at the hepatic lesion (SUVmax = 1.3) approaching the liver background (SUVmax = 1.1) (arrow). PARP-1 IHC staining of hepatic metastases showed low PARP-1 protein expression in tumor cells.

## 3 Discussion

Recent advances in AI-assisted and computer-aided drug design include interface-guided peptide binder design, applications in molecular imaging, and strategies for ligand discovery against traditionally challenging targets [[23], [24], [25]]. Integrated AI workflows for radiotracer development remain scarce, even as artificial intelligence is transforming radiopharmaceutical design and ligand discovery for undruggable targets. This study established a decision-support framework that integrates scaffold-constrained generative modeling and computational triage, and applied it to the development and exploratory clinical evaluation of a ^68^Ga-labeled PARP-1-targeted PET probe.

For this AI-driven prioritization system, we integrated similarity-based generative molecular design with a structure-aware, multi-objective screening strategy into a reproducible closed loop. We further validated the binding stability of key complexes by stress-testing them with molecular dynamics simulations. This workflow achieved a progression from AI proposal (001–007), convergence of synthesizability and structural rationality (005), radiochemically labelable precursors, and ^68^Ga-labeled radiotracers (NOTA/DOTA-FZPF). This multi-objective trade-off among affinity, synthesizability, and *in vivo* behavior provides a case study of AI-assisted radiotracer development. Crucially, structural optimization of radiotracers is highly sensitive to linker or chelate modifications; minor alterations in charge distribution or polarity can significantly impact plasma protein binding and clearance pathways, leading to decoupling between *in vitro* activity and *in vivo* imaging performance. This AI workflow, through CNN-and MD-based structural mechanism analysis, enables early identification and mitigation of such structural perturbation-pharmacokinetic amplification effects.

Optimization of *in vivo* biodistribution and target-to-background contrast remains a key consideration for the clinical translation of radiotracers [26]. Previous studies have described multiple PET radiotracers for imaging PARP-1 [27]. Notably, [^18^F]PARPi and [^18^F]FTT have undergone multiple clinical trials for in-depth investigation, with researchers continuously refining these compounds based on study results [14,15,[28], [29], [30]]. All currently known PARP-1 radiotracers exhibit high *in vivo* uptake in the abdominal cavity, particularly in the liver, spleen, kidneys, and intestines [31]. Such a physiological distribution may significantly impact lesion detection. This is because the abdomen is a common site of metastasis for malignancies such as breast, ovarian, prostate, and pancreatic cancers, where PARP inhibitors are used as first-line therapies. Therefore, enhancing tumor uptake of PARP-1 radiotracers while reducing peritoneal accumulation to improve the probe's target-to-background ratio at tumor sites is an urgent priority.

Using our established screening workflow, we generated [^68^Ga]Ga-DOTA-FZPF and [^68^Ga]Ga-NOTA-FZPF, two novel radiotracers that visualize PARP-1 expression. Both radiotracers in this work exhibit high stability and hydrophilicity. In vitro cell assays showed selective PARP-1 binding, consistent with *in vivo* micro-PET/CT observations. [^68^Ga]Ga-NOTA-FZPF was excluded from further investigation because of its low tumor uptake and high non-target organ accumulation. Excitingly, the newly designed [^68^Ga]Ga-DOTA-FZPF enhances tumor enrichment at 1 h post-administration, with an intratumoral uptake of 5.03 ± 0.77 %ID/g in tested models, while reducing non-specific uptake in non-tumor regions such as the peritoneum (tumor/small intestine: 6.53 ± 0.83, tumor/large intestine: 8.36 ± 0.30, tumor/liver: 1.38 ± 0.28, tumor/pancreas: 7.60 ± 2.19, tumor/stomach: 15.16 ± 6.83), showing high specificity for micro-PET/CT imaging. Micro-PET/CT imaging in the 22Rv1 tumor model showed that [^68^Ga]Ga-DOTA-FZPF can distinguish tumor tissue from muscle within 60 min, achieving a TMR of 14.80 ± 8.80, further supporting its potential for PARP-1 imaging. In summary, [^68^Ga]Ga-DOTA-FZPF is a promising radiotracer for imaging PARP-1 expression in tumors.

We further investigated whether [^68^Ga]Ga-DOTA-FZPF can act as a pharmacodynamic imaging probe to assess PARP inhibitor target engagement. Two tumor models exhibited comparable PARP-1 expression and *in vivo* radiotracer uptake. To avoid redundant work, we selected the 22Rv1 model for subsequent exploratory PET studies of fuzuloparib target binding. Results showed that although Fuzuloparib treatment had limited efficacy in BRCA wild-type 22Rv1-bearing mice, tumor uptake significantly decreased post-treatment. This pattern is most consistent with reduced tracer-binding availability during pharmacological occupancy by unlabeled Fuzuloparib. Makvandi et al.'s research indicates that PARP inhibitor sensitivity is directly correlated with PARP-1 protein expression levels. Therefore, exploring a noninvasive method to measure PARP expression directly is essential [28].

Several reports indicate that accurate measurement and monitoring of PARP expression *in vivo* can provide critical information for disease prognosis [32,33]. PARP expression is independently associated with poorer outcomes in breast, ovarian, and other tumors [[33], [34], [35]]. Assessing activation of DNA damage repair signaling may also help evaluate the effectiveness of genotoxic treatments following chemotherapy or radiotherapy.

The preliminary clinical study aimed to evaluate the feasibility and safety of this radiotracer for PET/CT imaging targeting PARP-1-overexpressing tumors. To date, no prior clinical investigation has been reported on the use of ^68^Ga-labeled PARP-1-targeting radiotracers, nor has there been any clinical evaluation of AI-designed radiotracers. This study thus reports a preliminary attempt to explore these two aspects. Four patients with breast or ovarian cancer were enrolled. [^68^Ga]Ga-DOTA-FZPF exhibits rapid blood clearance and non-specific organ clearance, primarily excreted via the kidneys, showing favorable pharmacokinetic properties. No adverse events or vital sign abnormalities were observed post-injection, confirming the good safety profile and tolerability of [^68^Ga]Ga-DOTA-FZPF in patients. In this limited exploratory cohort, one lesion from patient 1 with high PARP-1 expression showed higher tumor uptake (SUVmax = 3.7), whereas one lesion from patient 2 with low PARP-1 expression showed lower uptake (SUVmax = 1.3), offering preliminary reference information. Notably, compared with [^18^F]FTT and [^18^F]PARPi imaging results, the clinical trial data from this study indicated significantly reduced uptake in non-specific organs (e.g., liver, spleen, gastrointestinal tract, bones) despite insufficient tumor uptake of radioactivity [14,15]. Additionally, the limited sample size in this clinical study and the relatively low PARP-1 expression at the lesions among enrolled patients may have contributed to insufficient tumor uptake of [^68^Ga]Ga-DOTA-FZPF.

Furthermore, it should be noted that the pronounced hydrophilicity of the DOTA chelator may have reduced the amount of the radiotracer that effectively penetrates the cell membrane [36]. The modest tumor-to-liver ratio (1.38 ± 0.28) may limit the sensitivity of [^68^Ga]Ga-DOTA-FZPF for detecting hepatic metastases, a common site of metastasis in several solid tumors, and further optimization is warranted. Another limitation is that clinical studies include fewer cases and tumor types, potentially leading to biased results. In addition, the limited number of IHC samples precluded quantitative correlation analysis between PARP-1 expression and [^68^Ga]Ga-DOTA-FZPF SUVmax. Although evidence supports the feasibility of PET/CT imaging, further in-depth research is required to inform clinical trials and validate PET radiotracers for PARP-1.

### 3.1 Conclusion

In summary, this study established and validated an AI-assisted rational design workflow for developing PARP-1 radiolabeling probes. Under a unified decision-making framework, computational predictions and synthetic-tractability assessments were considered alongside medicinal chemistry judgment to support candidate selection for experimental evaluation. Based on this workflow, we systematically constructed two ^68^Ga-labeled radiotracers. [^68^Ga]Ga-DOTA-FZPF exhibited favorable pharmacokinetics, favorable PARP-1 targeting capabilities, high tumor uptake, low non-target organ retention in preclinical models, and the potential to enable real-time assessment of PARP inhibitor–target engagement during therapy. As an innovative radiotracer, [^68^Ga]Ga-DOTA-FZPF showed preliminary clinical value in a first-in-human study. These findings provide preliminary evidence for the use of [^68^Ga]Ga-DOTA-FZPF as an effective tool for noninvasively assessing the expression levels of PARP-1 in tumors. This study demonstrates how established AI and computational tools can be integrated with medicinal chemistry judgment and experimental evaluation to support radiotracer design and candidate prioritization. It provides a case study of AI-assisted radiotracer development for radiotracers targeting other molecules.

## 4 Experimental section

### 4.1 Materials and instrumentation

All common reagents or materials were purchased from commercial sources and used without further purification. Acetazolamide and trifluoroacetic acid (TFA) were obtained from Selleck Chemicals in Shanghai, China. Acetonitrile and ethanol were purchased from Macklin Biochemical Technology Co., Ltd.

NMR spectra were recorded on a Bruker AMX 400 spectrometer and calibrated using residual deuterated solvent as an internal reference (DMSO-*d*_6_: 2.5104 ppm for ^1^H NMR). All analytical MS data were recorded using Electron Spray Ionization (ESI).

The ^68^Ge - ^68^Ga generator was obtained from Eckert & Ziegler (Berlin, Germany). The radiopharmaceuticals were analyzed using radioactive reversed-phase high-performance liquid chromatography (radio-HPLC; Agilent 1260 Infinity II) and radioactive thin-layer chromatography (radio-iTLC; Dual Scan-RAM radio-TLC).

DOTA-FZPF and NOTA-FZPF were synthesized by the chemical company Co., Ltd. in Shanghai and analyzed via reverse-phase high-performance liquid chromatography (HPLC). The compounds DOTA-FZPF, NOTA-FZPF, [^68^Ga]Ga-DOTA-FZPF, and [^68^Ga]Ga-NOTA-FZPF were analyzed by the method: Kinetex 5 μm particles C-18 100A 250 * 4.6 mm (00G-4633-E0), 5 - 95 % acetonitrile (0.1 % TFA) in H_2_O (0.1 % TFA) in a linear gradient about 20 min, 1 mL/min, UV = 254 nm.

The Micro-PET/CT imaging studies were performed on an Inveon PET/CT (Siemens Medical Solutions) and reconstructed using Inveon Research (Siemens Medical Solutions). Radioactivity was measured with a γ-counter (SN-697, Shanghai Nuclear Institute Rihuan Photoelectric Instrument Company Limited).

### 4.2 Scaffold-constrained analog generation using Lib-INVENT (encoder–decoder RNN)

Using Fuzuloparib as the parent structure, scaffold-constrained analog generation was performed using the pretrained reaction-based Lib-INVENT RNN decorator model in inference mode. The pharmacophore-containing core was fixed, and the permitted substitution site was designated by a dummy attachment atom ([*]). Two independent decoration runs were performed using scaffold definitions with or without a retained methylene spacer, yielding compounds 001–003 and 004–007, respectively. For each analog, the scaffold SMILES, generated R-group, complete product SMILES, and model-derived negative log-likelihood (NLL) were recorded.

### 4.3 AI-based synthetic feasibility assessment using ASKCOS

We employed the ASKCOS platform developed by MIT to evaluate the synthetic feasibility of candidate compounds. We recorded the SCScore for each candidate and the availability of key intermediates/building blocks in the proposed retrosynthetic route (particularly commercially available fragments). Based on this, we prioritized lead structures with higher synthetic feasibility and suitability for further radiotracer derivatization.

### 4.4 Molecular docking studies

The human PARP-1 crystal structure (PDB ID: 5DS3) with a resolution of 2.60 Å was downloaded from the RCSB Protein Data Bank (PDB). The downloaded PARP-1 protein structures were then prepared, during which water molecules were removed and hydrogens were added. Subsequently, the ligand structures Compound 001, 002, 003, 004, 005 (NH_2_-FZPF), 006 (Fuzuloparib), 007, DOTA-FZPF, NOTA-FZPF, [^nat^Ga]Ga-DOTA-FZPF, and [^nat^Ga]Ga-NOTA-FZPF were converted to sdf format. Molecular docking was performed individually using the Gnina 1.0 (University of Pittsburgh, USA) to simulate interactions between each ligand and the PARP-1 protein. Rescore was selected as the CNN Scoring Mode. The docking results were analyzed using PyMOL (version 2.4.0, Schrödinger LLC, USA) and ChimeraX (version 1.11, USA), focusing on the three-dimensional binding modes and binding kinetics of PARP-1 with the ligands.

### 4.5 In silico target prediction and off-target risk evaluation based on a deep neural network (DNN) model

To assess the potential off-target interactions of the designed probes and verify their specificity toward PARP-1, an AI-driven reverse screening was performed using a deep neural network (DNN) model that captures both local substructural features and global molecular topology. The ECFP4+MHFP6 descriptors were trained on 7546 targets derived from the ChEMBL database, encompassing a diverse range of biological entities, including single proteins, protein complexes, protein families, cell lines, and organisms. During the prediction phase, the model simultaneously estimated activity probabilities for all targets, and the results were ranked by confidence score (0-1), reflecting the relative likelihood of interaction.

### 4.6 Molecular dynamics

All MD simulations were conducted using GROMACS 2022.2. The Amber14SB force field was used to parameterize the protein topology, and the TIP3P water model was chosen as the explicit solvent. Ligand topologies were generated using the Antechamber module with AM1-BCC partial charges and GAFF2 atom types, and subsequently converted to GROMACS-compatible formats using ACPYPE. Ion parameters compatible with the TIP3P model were derived from the Joung-Cheatham set. Each protein–ligand complex was solvated in a truncated dodecahedral box, ensuring a minimum distance of 1.2 nm between the solute surface and the box boundary. The systems were neutralized and adjusted to a physiological ionic strength of 0.15 M by adding Na^+^ and Cl^−^ counterions.

Energy minimization was performed using the steepest descent algorithm until the maximum force on any atom was less than 1000 kJ mol^−1^ nm^−1^. Equilibration was subsequently conducted in two phases: 200 ps of NVT (constant number of particles, volume, and temperature) followed by 200 ps of NPT (constant number of particles, pressure, and temperature) at 298 K. The production runs were carried out under NPT conditions for 100 ns using a time step of 2 fs and the Verlet cutoff scheme. Long-range electrostatic interactions were treated with the particle mesh Ewald (PME) method, while both Coulombic and van der Waals interactions were truncated at 1.2 nm. All bonds involving hydrogen atoms were constrained using the LINCS algorithm. Temperature and pressure were maintained at 298 K and 1 bar using the Nose-Hoover thermostat and Parrinello-Rahman barostat, respectively. Trajectory data were recorded at 10 ps intervals.

### 4.7 Radiolabeling and quality control

The radiolabeling of DOTA-FZPF ligand with the radionuclide Ga-68 was conducted employing the following protocol: Briefly, a volume of 1 mL of [^68^Ga]GaCl_3_, which was eluted from a^68^Ge/^68^Ga generator (IGG100, Eckert & Ziegler, Berlin, Germany), was brought into reaction with 10 μg of DOTA-FZPF in a 137 μL volume of 1.0 M sodium acetate buffer. This reaction was carried out under thermocycling conditions at 100°C for 10 min. The radiolabeling of NOTA-FZPF ligands under thermocycling conditions at 75°C was performed following an analogous protocol.

The radioactive product was purified before use. The final product was eluted from the C18 column by injecting 1.0 mL of aqueous ethanol (V_ethanol_: V_water_ = 70 %:30 %). The radiochemical purity was evaluated using radio-thin-layer chromatography (radio-iTLC) and radio-high-performance liquid chromatography (radio-HPLC).

### 4.8 Determination of the partition coefficient

Log P: An aliquot of each radiotracer (total activity 74 - 185 kBq) was dissolved in 1 mL of a biphasic solvent system (1-octanol: ultrapure water = 1:1). The mixture was vortex-mixed and centrifuged at 15,000 g for 10 min to achieve complete phase separation. Aliquots (100 μL) of the aqueous and organic phases were collected, and the radioactivity of each was measured using a calibrated γ-counter. Repeat five times.

Log D_7.4_: Similarly, mix a radiotracer with an activity of 74 - 185 kBq with 1 mL of solvent mixture (1-octanol: PBS = 1:1, PBS: pH 7.4). Follow the same measurement procedure described above. Five independent experimental repetitions were performed.

### 4.9 Radiochemical stability

*In vitro* stability: Mix [^68^Ga]Ga-DOTA-FZPF and [^68^Ga]Ga-NOTA-FZPF separately with 200 μL of PBS (pH 7.4) and incubate at 37°C for 3 h. At 1, 2, and 3 h, take an appropriate volume of the mixture for radio-HPLC quality control analysis. The experimental procedure for mouse serum (OMHD-E0093, Oumarsi) and human serum (GellCell U.S.) is the same as above.

*In vivo* stability: Administer 1.85 MBq of the radiotracer via tail vein injection in mice. After 3 h, an appropriate volume of mouse urine was collected for radio-HPLC detection.

Each of the above experiments was repeated three times.

### 4.10 Cellular culture

The AsPC-1, A2780, and 22Rv1 cell lines were all purchased from the Cell Resource Center of the Shanghai Institutes for Biological Sciences, Chinese Academy of Sciences (Shanghai, China). Cells were cultured in RPMI 1640 medium supplemented with 10 % fetal bovine serum and 1 % penicillin/streptomycin at 37°C in a 5 % CO_2_ incubator.

### 4.11 Tumor xenograft models

The study was granted by the Ethics Committee of Fudan University Shanghai Cancer Center (No. 2019 Cancer Research JS-147). 6 - 8 week-old male and female BALB/c nude mice were purchased from Jiangsu GemPharmatech Company for use in this study. To establish a tumor-bearing model (CDX), 5 × 10^6^ cells (100 μL) were subcutaneously inoculated into the right axillary region of the mice. When the tumor diameter reached 0.5 - 1 cm, the mice were ready for subsequent studies.

### 4.12 Western blot

Once the growth density of AsPC-1, A2780, and 22Rv1 cells reached approximately 80 %, the cells were detached and gathered for protein extraction. Cell lysates were carefully prepared in RIPA buffer containing protease inhibitor (P8340, Sigma Life Sciences) and phosphatase inhibitors 2 and 3 (P5726, P0044, Sigma Life Sciences). Quantification was performed using the BCA Protein Quantification Kit (Beyotime). Equal amounts of protein were electrophoresed and transferred to polyvinylidene difluoride membranes. Membranes were blocked at room temperature for 15 min with Rapid Blocking Solution (Epizyme). Membranes were then incubated overnight at 4°C with primary antibodies anti-PARP-1 (1:1000; ab191217, Abcam, Cambridge, UK) and GAPDH (1:1000; 2118, Cell Signaling Technology, Boston, USA). After three washes with TBST (Beyotime), membranes were incubated with horseradish peroxidase-conjugated secondary antibody (1:20,000; ab205718, Abcam, Cambridge, UK) at room temperature for 1 h. Finally, membranes were washed three times with TBST and incubated with Pierce ECL Blotting Substrate. Protein signals were detected using a LiCor Odyssey CLx imager (Lincoln, Nebraska).

### 4.13 Cellular uptake

Seed cells at a density of 2 × 10^5^ cells per well in a 24-well plate. Allow cells to attach for 24 h. Add 1 mL of radiotracer (74 kBq/mL) or Fuzuloparib (1 mg/mL) and continue incubation for 0.5, 1, and 2 h. Subsequently, wash cells twice with 500 μL PBS (pH 7.4, 0.01 M) and lyse with 500 μL NaOH (1 M). Collect lysates. Measure radioactivity using a γ-counter in counts per minute (CPM), applying decay correction based on injection time. Repeat the experiment three times at each time point.

### 4.14 Pharmacokinetics

The pharmacokinetic parameters of [^68^Ga]Ga-DOTA-FZPF and [^68^Ga]Ga-NOTA-FZPF were evaluated in normal mice. The radiotracer was injected into each mouse via the tail vein at a dose of 1.48 - 2.96 MBq in 200 μL. Blood samples were obtained via capillary tubes (specifications: 0.5×100; diameter: 0.5 mm; length: 100 mm) from the tail vein at 1, 5, 10, 15, 30, 60, 90, 120, and 180 min post-injection. Radioactivity was measured using a γ-counter and decay-corrected based on injection time. All samples were weighed. Radioactive CPM (counts per minute) was divided by capillary blood mass and expressed as %ID/g. Finally, pharmacokinetic parameters were analyzed using GraphPad Prism 9.4.0.

### 4.15 Micro-PET/CT imaging

AsPC-1, A2780, and 22Rv1 tumor-bearing mice (n = 3/group) received tail vein injections of a radiotracer (3.7 - 7.4 MBq/mouse). At 0.5, 1, and 2 h post-injection, mice were anesthetized with inhaled isoflurane and placed on an imaging bed for micro-PET/CT. Blockade experiments were performed in 22Rv1 and A2780 tumor models by co-injecting the radiotracer with an excess of Fuzuloparib (250 μg/mouse). Micro-PET and CT images were reconstructed using the 3D ordered-subsets expectation maximization (OSEM3D) algorithm and the maximum-value algorithm and fused in Inveon Research Workplace (Siemens Healthineers). Attenuation correction was subsequently applied. Doses were converted to the percentage of the injected dose per gram (%ID/g) of tissue. T/NT ratios were calculated as tumor uptake divided by contralateral muscle. Estimated absorbed doses for the heart, liver, muscle, and kidney were similarly derived from micro-PET data.

### 4.16 Ex vivo biodistribution (22Rv1, A2780, and AsPC-1 tumor models)

Biodistribution studies were conducted on 22Rv1, A2780, and AsPC-1 tumor-bearing mice (12 - 14 weeks old, n = 3/group). (200 μL; 1.85 - 5.55 MBq) was administered via tail vein injection. Three mice per group were euthanized at 0.5, 1, and 2 h post-injection, and target tissues were collected. Tissue radioactivity (tumor, blood, muscle, bone, liver, spleen, kidney, heart, lung, bladder, brain, intestine, pancreas, and stomach) was measured using a γ-counter, with radioactivity corrected for attenuation over time. Finally, tissue activity was calculated using GraphPad Prism 9.4.0 and expressed as %ID/g. This enabled further comparison and validation of [^68^Ga]Ga-DOTA-FZPF's specific uptake across different tumor models.

### 4.17 H&E staining and immunohistochemistry (IHC) staining

Tumor tissue from tumor-bearing mice was excised and sectioned into thin slices. The serial sections were stained with H&E (hematoxylin and eosin) and immunohistochemistry. Immunohistochemistry antibody information: anti-PARP-1 (1:1000 dilution; ab191217, Abcam, Cambridge, UK).

### 4.18 Acute tolerability assessment of radiotracers at radiotracer dose

[^68^Ga]Ga-DOTA-FZPF (3.7 - 7.4 MBq in 200 μL saline), [^68^Ga]Ga-NOTA-FZPF (3.7 - 7.4 MBq in 200 μL saline), or saline (200 μL) was injected into the tail vein of normal mice (n = 3 per group). Vital signs were monitored continuously for 7 days. Mice were euthanized at the end of the study to assess liver and kidney function and for H&E staining of the heart, liver, spleen, lungs, and kidneys.

### 4.19 PET assessment of fuzuloparib target engagement

To assess the receptor binding dynamics during the administration of Fuzuloparib in the 22Rv1 tumor model, [^68^Ga]Ga-DOTA-FZPF was used as a radiotracer. A total of 6 tumor-bearing mice with uniform tumor sizes were selected and randomly assigned to two distinct cohorts: the control group receiving saline (200 μL/mouse/day) and the treatment group receiving Fuzuloparib at 6 mg/kg once daily (n = 6 per group). A schematic representation of the experimental design is depicted in Fig. 6A.

The study duration was set at 21 days, with body weight and tumor volume monitored for each mouse throughout the study. Imaging via micro-PET/CT was conducted at four time points: on the first day (prior to treatment initiation) and on the 7th, 14th, and 21st days. Following the final imaging session on day 21, all mice were euthanized in accordance with ethical guidelines, and tumor tissues were excised for comprehensive immunohistochemistry (IHC) staining analyses.

### 4.20 Patients

This work is a small preliminary exploratory clinical observation enrolling only 4 patients. The human study received approval from the Ethics Committee of Fudan University Shanghai Cancer Center (number 2503316-22). The study was conducted in accordance with the principles outlined in the 1964 Declaration of Helsinki and its subsequent amendments. All participants signed written informed consent forms before they underwent [^68^Ga]Ga-DOTA-FZPF scans. Eligible patients were adults aged ≥18 years with tumors indicated for PARP inhibitor therapy according to clinical guidelines, including but not limited to breast and ovarian cancer. Patients receiving ongoing drug intervention, those with severe hepatic or renal dysfunction, and pregnant or lactating individuals were excluded. Three breast cancer patients and one ovarian cancer patient were included in this study. The radiotracer's capacity to identify PARP in patients was evaluated by measuring SUVmax (Standardized Uptake Value max), which reflects radiotracer uptake in normal organs and tumor tissues.

### 4.21 PET-CT scans and reconstruction protocols

[^68^Ga]Ga-DOTA-FZPF PET-CT examinations were performed using a Biograph mCT Flow scanner (Siemens Healthineers, Knoxville, TN, USA). After intravenous administration of a certain dose of [^68^Ga]Ga-DOTA-FZPF (Table S3), patients rested in a quiet environment for approximately 60 min before image acquisition. CT data were acquired at 120 kV with automated tube current adjustment, utilizing a slice thickness of 3 mm, a slice increment of 2 mm, a pitch factor of 1.0, and a gantry rotation duration of 0.5 s. PET data acquisition lasted 2-3 min for each scanning bed. Iterative image reconstruction of PET datasets was implemented using the ordered subset expectation maximization algorithm, with 2 reconstruction iterations and 21 subsets, to generate a 200 × 200 matrix for subsequent quantitative measurements. The maximum standardized uptake value (SUVmax) was calculated to quantify intratumoral radiotracer accumulation of [^68^Ga]Ga-DOTA-FZPF.

### 4.22 Statistical analysis

All quantitative data are expressed as mean ± standard deviation (SD). All statistical analyses were performed using GraphPad Prism (version 9.4.0). Independent samples t-tests and Mann-Whitney U tests were used to compare results between groups. Values of P < 0.05 were considered statistically significant.

## CRediT authorship contribution statement

**Mengjing Ji:** Writing – original draft, Methodology, Formal analysis. **Chunjuan Jiang:** Writing – original draft. **Yue Xi:** Methodology, Data curation. **Shuai Liu:** Investigation, Data curation. **Simin He:** Data curation. **Jianping Zhang:** Methodology. **Xiaoping Xu:** Investigation. **Zhongyi Yang:** Writing – review & editing, Project administration, Methodology, Investigation. **Xiangwei Wang:** Writing – review & editing, Project administration, Methodology, Funding acquisition, Conceptualization. **Shaoli Song:** Writing – review & editing, Project administration, Conceptualization.

## Ethics

This study was approved by the Ethics Committee of Fudan University Shanghai Cancer Center (No. 2503316-22), and written informed consent was obtained from the patient.

## Declaration of competing interest

The authors declare that they have no known competing financial interests or personal relationships that could have appeared to influence the work reported in this paper.

## Data Availability

Data will be made available on request.
