# Supplementary material for: An artificial intelligence-assisted PARP-1-targeted PET imaging agent [68Ga]Ga-DOTA-FZPF: From bench to clinic
Source: Mater Today Bio. 2026 Oct 7;41:103728. doi: 10.1016/j.mtbio.2026.103728 (PMC13678008; doi:10.1016/j.mtbio.2026.103728)
Supplement: Supplementary file 1 [file mmc1.doc]

**Supplemental Material**

**An Artificial Intelligence-Assisted PARP-1-Targeted PET Imaging Agent [^68^Ga]Ga-DOTA-FZPF: From Bench to Clinic**

Mengjing Ji ^#a,b,c,d^, Chunjuan Jiang ^#a,b,c,d^, Yue Xi ^a,b,c,d^, Shuai Liu ^a,b,c,d^, Simin He ^a,b,c,d^, Jianping Zhang ^a,b,c,d^, Xiaoping Xu ^a,b,c,d^, Zhongyi Yang ^***a,b,c,d^, Xiangwei Wang ^**a,b,c,d^, Shaoli Song ^*a,b,c,d^

^a^ Department of Nuclear Medicine, Fudan University Shanghai Cancer Center, Shanghai 200032, China

^b^ Department of Oncology, Shanghai Medical College, Fudan University, Shanghai 200032, China.

^c^ Center for Biomedical Imaging, Fudan University, Shanghai 200032, China

^d^ Shanghai Engineering Research Center of Molecular Imaging Probes, Shanghai 200032, China

^#^ First authors contributed equally to this work

Mengjing Ji, E-mail: jimengjing98@163.com;

Chunjuan Jiang, E-mail: tsyxy2004@163.com;

^***^ Corresponding authors: Zhongyi Yang, MD, yangzhongyi21@163.com

^**^ Corresponding authors: Xiangwei Wang, MD, Xiangweiwang2023@outlook.com

^*^ Corresponding authors: Shaoli Song, MD, **the lead contact**, [shaoli-song@163.com](mailto:shaoli-song@163.com)

**Table S1. Deep learning (CNN)-guided docking evaluation for Fuzuloparib, Fuzuloparib analogs, NOTA-FZPF, DOTA-FZPF, [^nat^Ga]Ga-NOTA-FZPF, and [^nat^Ga]Ga-DOTA-FZPF interactions with PARP-1 (CNN pose score and CNN affinity).**

| Compounds and PARP-1 | Affinity (kcal/mol) | Intramol (kcal/mol) | CNN Pose Score | CNN Affinity |
| --- | --- | --- | --- | --- |
| 001 | -12.99 | -0.78 | 0.9842 | 7.46 |
| 002 | -13.39 | -0.78 | 0.9758 | 7.318 |
| 003 | -12.77 | -0.89 | 0.9838 | 7.513 |
| 004 | -13.32 | -0.77 | 0.9836 | 7.355 |
| 005 (NH_2_-FZPF) | -12.34 | -0.95 | 0.7815 | 7.044 |
| 006 (Fuzuloparib) | -13.47 | -1 | 0.9782 | 7.505 |
| 007 | -12.14 | -1.06 | 0.8157 | 7.265 |
| NOTA-FZPF | -11.76 | 0.48 | 0.8964 | 7.651 |
| DOTA-FZPF | -11.26 | 0.9 | 0.3889 | 7.309 |
| [^nat^Ga]Ga-NOTA-FZPF | -11.76 | -2.61 | 0.5067 | 7.385 |
| [^nat^Ga]Ga-DOTA-FZPF | -11.24 | -0.24 | 0.4673 | 7.278 |

**Table S2. Deep neural network (DNN)-driven target prediction for Fuzuloparib, NH_2_-FZPF, NOTA-FZPF, and DOTA-FZPF (confidence score, rank, and target annotations).**

| Compound | Target Name | Confidence Score | Rank | Target Protein Class | Target Organism | Target Type |
| --- | --- | --- | --- | --- | --- | --- |
| Fuzuloparib | PARP-1 | 1 | 1 | enzyme transferase | Homo sapiens | SINGLE PROTEIN |
| NH_2_-FZPF | PARP-1 | 0.98 | 1 | enzyme transferase | Homo sapiens | SINGLE PROTEIN |
| NOTA-FZPF | PARP-1 | 1 | 1 | enzyme transferase | Homo sapiens | SINGLE PROTEIN |
| DOTA-FZPF | PARP-1 | 1 | 1 | enzyme transferase | Homo sapiens | SINGLE PROTEIN |

**Table S3. Information on representative patients enrolled in PET-CT imaging studies using [^68^Ga]Ga-DOTA-FZPF.**

| Patient  No. | Age  (Years old) | Weight  (Kg) | Diagnosis | HRD Status | Histologic Subtype | [^68^Ga]Ga-DOTA-FZPF PET/CT (MBq) | [^68^Ga]Ga-DOTA-FZPF PET-CT protocol |
| --- | --- | --- | --- | --- | --- | --- | --- |
| 1 | 24 | 65 | Breast cancer | Positive | IDC-NOS | 167.98 | Static (50-70 min) |
| 2 | 52 | 64 | Breast cancer | Positive | IDC-NOS | 159.14 | Static (50-70 min) |
| 3 | 45 | 66 | Ovarian cancer | Positive | HGSC | 169.09 | Static (50-70 min) |
| 4 | 33 | 80 | Breast cancer | Not assessed | IDC-NOS | 126.91 | Static (50-70 min) |

Abbreviations: IDC-NOS, Invasive ductal carcinoma, not otherwise specified; HGSC, high-grade serous carcinoma; HRD, homologous recombination deficiency.

**Table S4. SUVmax of each organ and normal tissue in every patient.**

| Target Organ | SUVmax (Patient 1) | SUVmax (Patient 2) | SUVmax (Patient 3) | SUVmax (Patient 4) |
| --- | --- | --- | --- | --- |
| Heart | 2.7 | 1.9 | 3.7 | 1.9 |
| Liver | 1.2 | 1.1 | 1.4 | 1.2 |
| Spleen | 1.2 | 1.7 | 2.2 | 0.9 |
| Lung | 0.5 | 0.5 | 0.8 | 0.7 |
| Kidney | 57.6 | 7.6 | 21.4 | 5.3 |
| Stomach wall | 0.4 | 0.4 | 1.2 | 0.4 |
| Small intestine | 1.3 | 0.6 | 1.8 | 0.6 |
| Large intestine | 0.9 | 0.3 | 0.6 | 0.9 |
| Muscle | 0.4 | 0.3 | 0.6 | 0.3 |
| Ovaries | 2.3 | 1.2 | 3.1 | 0.8 |
| Uterus | 2.4 | 1.7 | 1.8 | 1.8 |
| Pancreas | 0.9 | 1.2 | 1.9 | 1.0 |
| Brain | 0.5 | 0.1 | 0.6 | 0.1 |
| Breast | 1.8 | 1.1 | 1.3 | 1.4 |
| Bone | 0.3 | 0.3 | 0.6 | 0.9 |
| Thyroid | 1.6 | 0.8 | 1.8 | 0.8 |

**Figure S1. Controlled expansion of Fuzuloparib analogs (001–007) via SMILES-based encoder-decoder RNN chemical language model.**

**Figure S2.** **Structures and analytical validation of DOTA-FZPF and NOTA-FZPF radiotracer precursors and their ^68^Ga-labeled counterparts.** (A) Chemical structure of the compounds DOTA-FZPF, NOTA-FZPF, [^68^Ga]Ga-DOTA-FZPF, and [^68^Ga]Ga-NOTA-FZPF. (B) Radiochemical purity of the [^68^Ga]Ga-DOTA-FZPF and [^68^Ga]Ga-NOTA-FZPF. (C) HPLC spectra of DOTA-FZPF (left) and NOTA-FZPF (right). (D) Radio-HPLC spectra of [^68^Ga]Ga-DOTA-FZPF (left) and [^68^Ga]Ga-NOTA-FZPF (right).

**Figure S3. Preliminary acute tolerability assessment of the radiotracers at the tracer dose.** (A) Red blood cell (RBC), white blood cell (WBC), and platelet (PLT) count results for control (saline) and experimental groups injected with either [^68^Ga]Ga-DOTA-FZPF or [^68^Ga]Ga-NOTA-FZPF (left). Serum biochemical parameters assessing liver and kidney function, including alanine aminotransferase (ALT), aspartate aminotransferase (AST), alkaline phosphatase (ALP), blood urea nitrogen (BUN), and creatinine (CREA) count results for control (saline) and experimental groups injected with [^68^Ga]Ga-DOTA-FZPF or [^68^Ga]Ga-NOTA-FZPF (right). (B) H&E staining of the major organs from normal mice in control and experimental groups at 7 days post-injection. (n = 3)

**Figure S4.** **Preclinical evaluation of [^68^Ga]Ga-NOTA-FZPF radiotracer by microPET/CT imaging of the AsPC-1, A2780, and 22Rv1 tumor models.** MicroPET/CT imaging (A), quantification of regions of interest (B), and the tumor-to-muscle ratio (C) of [^68^Ga]Ga-NOTA-FZPF in A2780 models. MicroPET/CT imaging (D), quantification of regions of interest (E), and the tumor-to-muscle ratio (F) of [^68^Ga]Ga-NOTA-FZPF in the AsPC-1 models. Ex vivo biodistribution (G) and tumor-to-nontarget tissue ratio analysis (H) of [^68^Ga]Ga-NOTA-FZPF in the 22Rv1 model.

**Figure S5. PET/CT images of patients 3 and 4.** (A) [^68^Ga]Ga-DOTA-FZPF PET/CT shows radioactive uptake in the Ovarian cancer of Patient 3, who was diagnosed with ovarian cancer (SUVmax = 3.1) (arrow). (B) [^68^Ga]Ga-DOTA-FZPF PET/CT shows radioactive uptake in the left breast of Patient 4, who was diagnosed with breast cancer (SUVmax = 4.2) (arrow).

**NMR and MS spectra for compounds**

**The following are the data of compound characterization:**

**DOTA-FZPF:**

^1^H NMR (400 MHz, DMSO) δ 12.59 (s, 1H), 8.26 (d, *J* = 8.2 Hz, 2H), 7.97 (s, 1H), 7.90 (s, 1H), 7.83 (t, *J* = 7.5 Hz, 1H), 7.47 (s, 2H), 7.27 (d, *J* = 9.5 Hz, 1H), 4.73 (s, 1H), 4.34 (s, 2H), 4.11 (s, 1H), 3.98 (s, 1H), 3.85 (s, 2H), 3.66 (s, 2H), 3.48 (s, 10H), 2.96 (s, 16H), 2.00 (d, *J* = 7.7 Hz, 2H).

MS-ESI(+) calcd for C_39_H_49_FN_12_O_9_: 848.3729, [M]/2+1 found 425.4, [M+H]^+^ found 849.4.
HPLC t_R_ = 7.52 min.

**NOTA-FZPF:**

^1^H NMR (400 MHz, DMSO) δ 12.59 (s, 1H), 8.36 (s, 1H), 8.26 (d, *J* = 7.9 Hz, 1H), 7.97 (d, *J* = 7.8 Hz, 1H), 7.90 (t, *J* = 7.7 Hz, 1H), 7.84 (t, *J* = 7.4 Hz, 1H), 7.47 (d, *J* = 7.3 Hz, 2H), 7.28 (t, *J* = 8.9 Hz, 1H), 6.15 (s, 1H), 4.73 (s, 1H), 4.36 (d, *J* = 14.4 Hz, 2H), 4.14 – 3.64 (m, 5H), 3.54 (s, 6H), 3.23 (d, *J* = 24.1 Hz, 6H), 2.92 (d, *J* = 28.0 Hz, 12H).

MS-ESI(+) calcd for C_35_H_42_FN_11_O_7_: 747.3253, [M]/2+1 found 375.0, [M+H]^+^ found 748.4.

HPLC t_R_ = 8.08 min.

**Figure S6.** ^1^H NMR of compound **DOTA-FZPF**.

**Figure S7.** MS spectrum of compound **DOTA-FZPF**.

**Figure S8.** ^1^H NMR of compound **NOTA-FZPF**.

**Figure S9**. MS spectrum of compound **NOTA-FZPF**.
